# Identification of *PIK3CA* multigene mutation patterns associated with superior prognosis in stomach cancer

**DOI:** 10.1186/s12885-021-08115-w

**Published:** 2021-04-07

**Authors:** Yu Yu, Zhuoming Xie, Mingxin Zhao, Xiaohua Lian

**Affiliations:** 1 Department of Cell Biology, Basic Medical School, Army Medical University (Third Military Medical University), Chongqing, 400038 People’s Republic of China; 2 Beijing Syngentech Co., Ltd, Zhongguancun Life Science Park, Changping District, Beijing, 102206 People’s Republic of China; 3 State Key Laboratory of Proteomics, Beijing Proteome Research Center, Beijing Institute of Lifeomics, National Center for Protein Sciences (The PHOENIX Center, Beijing), Beijing, 102206 People’s Republic of China

**Keywords:** *PIK3CA* mutation, Higher-order genetic interaction, Marginal effect, Stomach adenocarcinoma, Cancer prognosis

## Abstract

**Background:**

*PIK3CA* is the second most frequently mutated gene in cancers and is extensively studied for its role in promoting cancer cell resistance to chemotherapy or targeted therapy. However, *PIK3CA* functions have mostly been investigated at a lower-order genetic level, and therapeutic strategies targeting *PIK3CA* mutations have limited effects. Here, we explore crucial factors interacting with *PIK3CA* mutations to facilitate a significant marginal survival effect at the higher-order level and identify therapeutic strategies based on these marginal factors.

**Methods:**

Mutations in stomach adenocarcinoma (STAD), breast adenocarcinoma (BRCA), and colon adenocarcinoma (COAD) samples from The Cancer Genome Atlas (TCGA) database were top-selected and combined for Cox proportional-hazards model analysis to calculate hazard ratios of mutation combinations according to overall survival data and define criteria to acquire mutation combinations with considerable marginal effects. We next analyzed the *PIK3CA + HMCN1 + LRP1B* mutation combination with marginal effects in STAD patients by Kaplan-Meier, transcriptomic differential, and KEGG integrated pathway enrichment analyses. Lastly, we adopted a connectivity map (CMap) to find potentially useful drugs specifically targeting *LRP1B* mutation in STAD patients.

**Results:**

Factors interacting with *PIK3CA* mutations in a higher-order manner significantly influenced patient cohort survival curves (hazard ratio (HR) = 2.93, *p-value* = 2.63 × 10^− 6^). Moreover, *PIK3CA* mutations interacting with higher-order combination elements distinctly differentiated survival curves, with or without a marginal factor (HR = 0.26, *p-value* = 6.18 × 10^− 8^).

Approximately 3238 *PIK3CA-*specific higher-order mutational combinations producing marginal survival effects were obtained. In STAD patients, *PIK3CA + HMCN1* mutation yielded a substantial beneficial survival effect by interacting with *LRP1B* (HR = 3.78 × 10^− 8^, *p-value* = 0.0361) and *AHNAK2* (HR = 3.86 × 10^− 8^, *p-value* = 0.0493) mutations. We next identified 208 differentially expressed genes (DEGs) induced by *PIK3CA + HMCN1* compared with *LRP1B* mutation and mapped them to specific KEGG modules. Finally, small-molecule drugs such as geldanamycin (connectivity score = − 0.4011) and vemurafenib (connectivity score = − 0.4488) were selected as optimal therapeutic agents for targeting the STAD subtype with *LRP1B* mutation.

**Conclusions:**

Overall, *PIK3CA*-induced marginal survival effects need to be analyzed. We established a framework to systematically identify crucial factors responsible for marginal survival effects, analyzed mechanisms underlying marginal effects, and identified related drugs.

**Supplementary Information:**

The online version contains supplementary material available at 10.1186/s12885-021-08115-w.

## Background

The phosphatidylinositol 3′-kinase (PI3K) signaling pathway is one of the most frequently altered pathways in human cancers that impacts major hallmarks of malignancies [1] and remarkably contributes to cancer initiation [2, 3], progression, metastasis, metabolism, and cell survival [4–8]. Aberrant PI3K signaling activity mostly accounts for the poor outcomes and tumor relapse seen in cancer patients [7] [9–11]. *PIK3CA*, a member of the PI3K family, encodes the p110α protein, and the p110α protein is a subunit within the PI3K catalytic domain [12]. Hotspot mutations of *PIK3CA* were found in a broad scope of cancers, including melanoma, breast cancers, colorectal cancers, gastric cancers, liver cancers, etc. [4, 13], and most of these mutations have been confirmed to promote an oncogenic gain-of-function effect. A considerable fraction (approximately 10 ~ 30%) of cancer patients carry abnormally activated *PIK3CA* mutants [8, 14]. These *PIK3CA* mutants enabled reduced dependence on growth factors, anchorage-independent growth, and enhanced resistance to apoptosis [15–17].

Interestingly, we noted that *PIK3CA* mutants cooccurred with other genetic alterations that promote enhanced PI3K signaling [18, 19]; for example, mutations in *PIK3CA* were found concomitantly with mutations in *EGFR, KRAS*, or *ALK* in the same tumors of lung cancer patients [5]. However, considering the complex genetic alteration context in cancer and the complex traits leading to higher-order mutational interactions, paired mutations might lack sufficient accuracy and robustness for precise applications in diagnosis and prognosis. Thus, a complete investigation of the genetic factors in higher-order combinations could be key to improving prognostic accuracy.

Intriguingly, in economics, the benefit experienced when adding one extra unit is called the marginal benefit [20]. We found that some lower-order gene mutants (we referred to lower-order mutants as those involved in combinations of fewer than three elements), defined as marginal factors in this study, could substantially affect the overall survival (OS) probability when combined with other specific cooccurring mutation combinations, defined as seed combinations, in a higher-order interaction (we referred to higher-order factors as those involved in combinations of equal to or more than three) (Supplementary Fig. 1). The effects of such higher-order combinations were categorized as higher-order genetic marginal effects. This study focused on the contributions of *PIK3CA* mutants to higher-order genetic marginal effects on the hazard ratio (HR) and overall survival (OS) probability across three different cancer types. Therefore, we established a *PIK3CA*-based subtype classification based on the frequencies of *PIK3CA* pairing partners to decisively identify marginal factors affecting the HR of the original mutational combination, attempted to reveal the mechanistic differences between these subtypes, and identified various small-molecule drugs according to the unique *PIK3CA* subtype signatures. To the best of our knowledge, this is the first study to systematically investigate the functional properties of *PIK3CA* from a higher-order point of view. Thus, this study provides a better understanding of the mutually interacting relationships between specific higher-order combinatorial mutations and *PIK3CA* mutants across a wide variety of cancer types and provides a framework to rationally identify and exploit factors interacting with *PIK3CA* mutations to induce a marginal effect.

## Methods

### Obtaining the clinical and genetic data

Somatic mutation (single-nucleotide polymorphism (SNP) or insertion/deletion (INDEL)), transcriptomic gene expression, and clinical data of breast adenocarcinoma (BRCA), colon adenocarcinoma (COAD), and stomach adenocarcinoma (STAD) patients were obtained from The Cancer Genome Atlas (TCGA) database (https://www.cancer.gov/about-nci/organization/ccg/research/structural-genomics/tcga) and the UCSC Xena database (https://xenabrowser.net/datapages/?hub=https://tcga.xenahubs.net:443).

### Identifying the genetic combinations that could impact the survival outcomes of cancer patients

Somatic mutation (SNP and INDEL), survival, and clinical datasets from BRCA, COAD, and STAD patients were extracted from the TCGA database. To evaluate the HR of the genetic combinations consisting of the *PIK3CA* mutants, we removed the “silent” mutations and selected the 35 most frequently occurring mutational genes by adopting a Cox proportional hazard regression model.

The original dataset samples were split: 60% of samples served as the exploration set, and 40% of samples served as the validation set. Then, the samples were further processed into the.maf format via the maftools package. Next, we selected the thirty-five highest ranked mutated genes from BRCA, COAD, and STAD patients as the research objects, enumerated the potential combinations that may contain one to six mutated genes, and calculated the HR and *p-value* through Cox hazard ratio modeling via the survGroup() function from the maftools package [21]; this analysis identified the impacts of the genetic sets on patient OS time and status.

### Discovering genes that caused significant marginal effects on the hazard ratio

The combinatorial gene sets of different orders were crossed and merged according to the shared gene names, and the conditions used to acquire the key gene that was causing the marginal effect on HR are listed as follows:

1) The *p-value* of the relative lower-order combination is larger than 0.3, while the *p-value* of the relative higher-order combination is lower than 0.01.

2) The HR of the relative lower-order combination is larger than 1. In comparison, the HR of the relatively higher-order combination was two times larger than the HR of the relatively lower-order combination, and the *p-value* of the relatively higher-order combination was lower than 0.05.

3) The HR of the relatively lower-order combination is larger than 1, while the HR of the relatively higher-order combination is lower than 1, and the *p-value* of the relatively higher-order combination is lower than 0.05.

The mutational combinations that generated significant marginal effects were ranked according to the fold-change of the higher-order HR against the lower-order HR, and the results were uploaded to the Synapse repository: https://www.synapse.org/#!Synapse:syn23530651.

### Plotting the Kaplan-Meier survival curve

The Kaplan-Meier survival curves were plotted using the surv_fit() function from the survminer package and the mafSurvival() function from the maftools package in the R 4.0.2 platform. These analyses were used to demonstrate the survival probability of patient cohorts with different mutational combinations. The *p-value* was calculated by the log-rank test, and the HR was calculated from a Cox proportional hazard model.

### Classifying the marginal effect-specific *PIK3CA* subtypes of BRCA, COAD, and STAD patients

Gene mutations that paired with *PIK3CA* mutations to induce a marginal effect and the original higher-order combinatorial constituents were summarized statistically in an individual cancer type. The genes within the same gene family were considered the same mutant type. We determined the belonging of genes to a gene family through the 35 selected genes by summarizing their common prefix characteristics. Genes with the same prefix characteristics were considered to belong to a gene family. The partners of the *PIK3CA* mutations that had the highest frequencies were chosen as the standards for candidate subtype classification.

### RNA differential expression analysis

Since the transcriptomic expression data (TCGA Stomach Cancer (STAD): IlluminaHiSeq UNC) we obtained from the Xena UCSC were as in log2(x + 1) transformed RSEM normalized count, we then restored the integer type of RSEM normalized read counts by round (2^log2(x + 1)^) method to meet the requirement that the input data should be an integer type, when using DESeqDataSetFromMatrix() from DESeq2 package, and transformed them into DESeq2 data through the DESeq2 package, and STAD patients with *LRP1B* mutation but without *PIK3A + HMCN1* comutation were chosen as the reference group to be compared with STAD patients with the *LRP1B + PIK3CA + HMCN1* trimutation. The data were then entered into the geom_point() function of the ggplot2 package for further plotting tasks; the fold-change threshold was ±2, and multiple test correction was applied based on an adjusted P cutoff value of 0.0001.

### Integrating the KEGG pathway-based data

The DEGs between the *LRP1B* without *PIK3CA + HMCN1* patient cohort and the *LRP1B* + *PIK3CA + HMCN1* cohort were obtained with a fold-change threshold of ±2 and a *p-value* threshold of 0.01. Next, the gene symbols of the DEGs were entered into EntrezID, and the fold-change values of the DEGs were calculated through the clusterProfiler package in the R 4.0.2 platform via the compareCluster (fun = ‘enrichKEGG’) function.

### Generating a connectivity map for selecting marginal factor-targeting compounds

To determine which small compounds might be effective against *LRP1B* mutation to induce a marginal beneficial effect, we entered 107 genes upregulated in the *LRP1B* single-mutation samples with poor prognosis compared with the *LRP1B + PIK3CA + HMCN1* trimutation samples with the Broad Institute’s Connectivity Map [22, 23], a public online tool (https://clue.io) (with registration), which enabled us to select the molecular compounds that can activate or inhibit the specific biological processes underlying each gene expression signature. The signature strength, replicative correlation (75th percentile), transcriptional activity score, and connectivity score thresholds were 200, 0.2, 0.2, and − 0.3, respectively. We next determined the compound signature strength and replicative correlation (75th percentile), transcriptional activity score, and connectivity score by using the scatter plot function ggplot2.

## Results

### Systematic analysis of multigene mutation patterns in BRCA, COAD, and STAD cancer samples

**Fig. 1:**
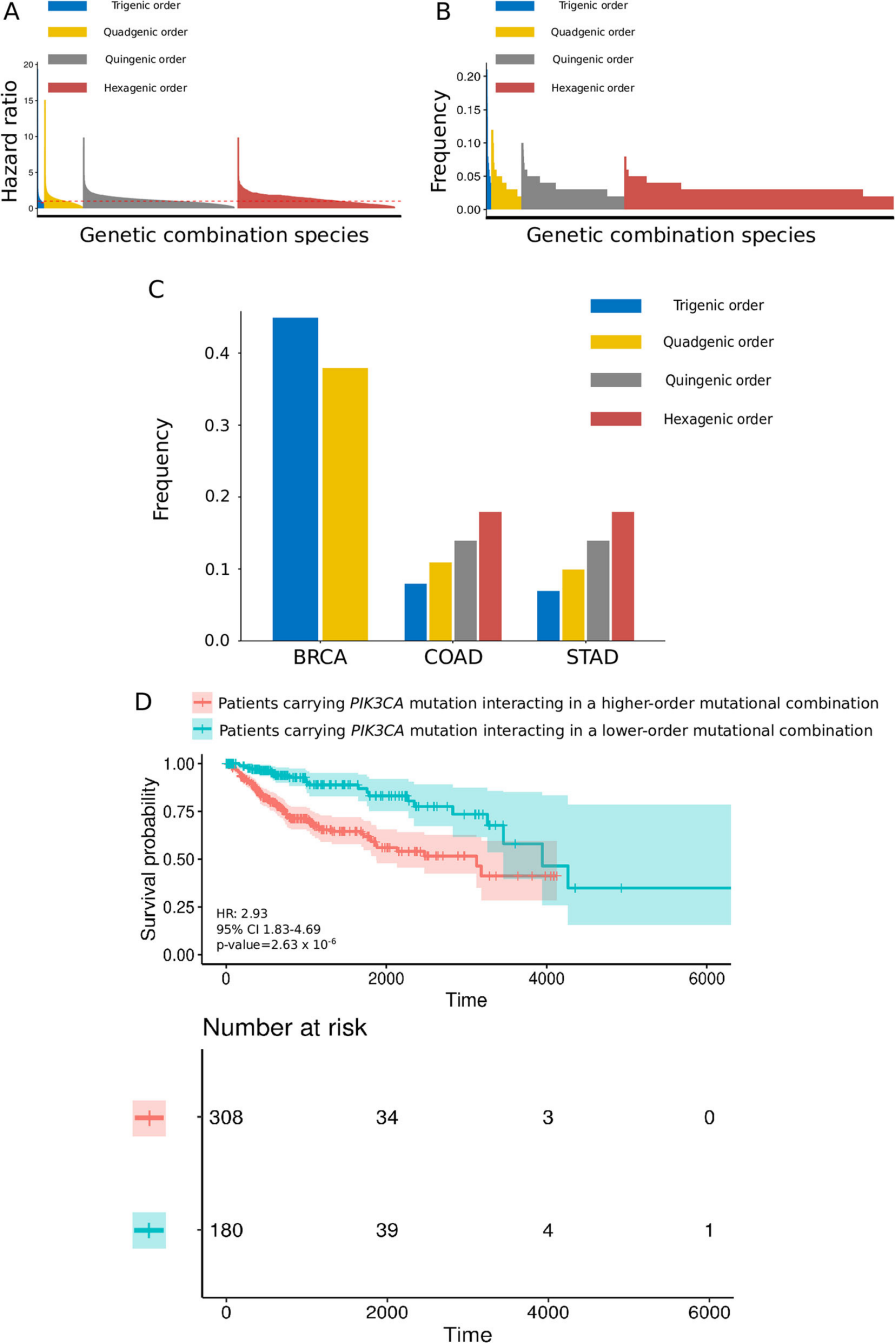
Systematic enumeration of higher-order mutational combinations within multiple genetic orders from STAD, BRCA, and COAD patient cohorts. **a** Plot of the hazard ratios for all combinatorial gene sets. The x-axis denotes the genetic combination species. The y-axis denotes the log10 (hazard ratio) + 1 value. The red dashed line distinguishes the adverse and beneficial effect zones. **b** Plot of coverage for all combinatorial gene sets. The x-axis denotes the genetic combination species. The y-axis denotes the relative proportion (%). **c** The proportion of *PIK3CA* mutations within the multiplexed combinations in each tumor type. The x-axis denotes the species of genetic combination. The y-axis denotes the relative proportion (%). **d** Kaplan-Meier survival curves of two patient cohorts from STAD, BRCA, and COAD patients carrying *PIK3CA* mutations presented in lower-order or higher-order mutational gene sets. The significance of the gene sets for predicting survival was calculated by using the log-rank test. The x-axis denotes the survival time. The y-axis denotes the survival probability

To identify multigene mutation patterns that have the potential to influence the survival of BRCA, COAD, and STAD patients, we selected the top 35 most frequently mutated genes comprising missense mutations, nonsense mutations, and INDELs from the TCGA BRCA, COAD, and STAD cancer cohorts; the cohorts included 911 patients in total. Since the genetic interaction orders represent the quantity of genetic elements within a specific genetic combination and this specific genetic combination might influence certain phenotypes, such as overall survival outcomes and overall survival time, we screened and found 11,782, 60,480, 205,723, and 464,519 unique species of higher-order combinations of the trigenic, quadgenic, quingenic, and hexagenic genetic orders, respectively. We found that the quantity of combination species increased as the genetic order increased (Fig. 1a). On the other hand, the combination coverage of cancer samples was increased as the genetic order decreased (Fig. 1b). These results indicated that the detailed study of higher-order genetic combinations may help understand cancer heterogeneity regarding variations in malignant states and would improve diagnostic, prognostic and therapeutic precision due to the high-resolution dissection of genetic diversity.

Next, we extracted and summarized the combinations containing *PIK3CA* mutations of trigenic to hexagenic order and found that more than 30–40% of the trigenic and quadgenic combinations contained *PIK3CA* mutations in BRCA patients, and 10–20% of trigenic to hexagenic combinations contained *PIK3CA* mutations in COAD and STAD patients (Fig. 1c). Interestingly, the combinations containing mutations of *PIK3CA* increased simultaneously with the genetic order in the COAD and STAD patient cohorts, which implied that *PIK3CA* may serve as a hub gene in complex genetic backgrounds. To ascertain the biological value of analyzing the higher-order genetic interaction that included *PIK3CA*, we first integrated the BRCA, COAD, and STAD patients carrying the *PIK3CA* mutation and categorized these patients into two cohorts. The criteria of categorizing these patients were dependent on the number of *PIK3CA* cooccurring mutated genes among the 35 top ranked, most frequently mutated genes. If the number of *PIK3CA* cooccurring mutated genes among the 35 candidate genes was none or 1, we then categorized this sort of patient as “patients carrying *PIK3CA* mutations interacting in lower-order mutational combinations”; if the number of *PIK3CA* cooccurring mutated genes among the 35 candidate genes was more than 1, we then categorized this sort of patient as “patients carrying *PIK3CA* mutations interacting in higher-order mutational combinations”. We observed that BRCA, COAD, and STAD patients carrying *PIK3CA* mutations in higher-order mutational combinations had a worse prognosis than BRCA, COAD, and STAD patients carrying *PIK3CA* mutations in only lower-order combinations (308 patients vs. 180 patients; HR 2.93, 95% confidence interval (CI) 1.83–4.69, *p-value* = 2.63 × 10^− 6^) (Fig. 1d), implying that efforts to study the potential interactions of *PIK3CA* mutations within higher-order combinations are warranted to improve the knowledge of *PIK3CA* mutations with their related mutations.

### *PIK3CA* mutations can produce meaningful marginal beneficial/adverse effects on survival outcomes within specific mutational combinations

This analysis was inspired by the marginal analysis concept, which studies how the quantified HR of higher-order combinations changes with an alteration in a singlegenic or a digenic gene set in lower-order combination. Therefore, we set any singlegenic or digenic mutation that promoted a remarkable survival effect in a completely higher-order combination as a marginal factor (Supplementary Fig. 1). The mutations that combined with the marginal factor to constitute the complete higher-order combination were taken as seed mutations (Supplementary Fig. 1).

**Fig. 2:**
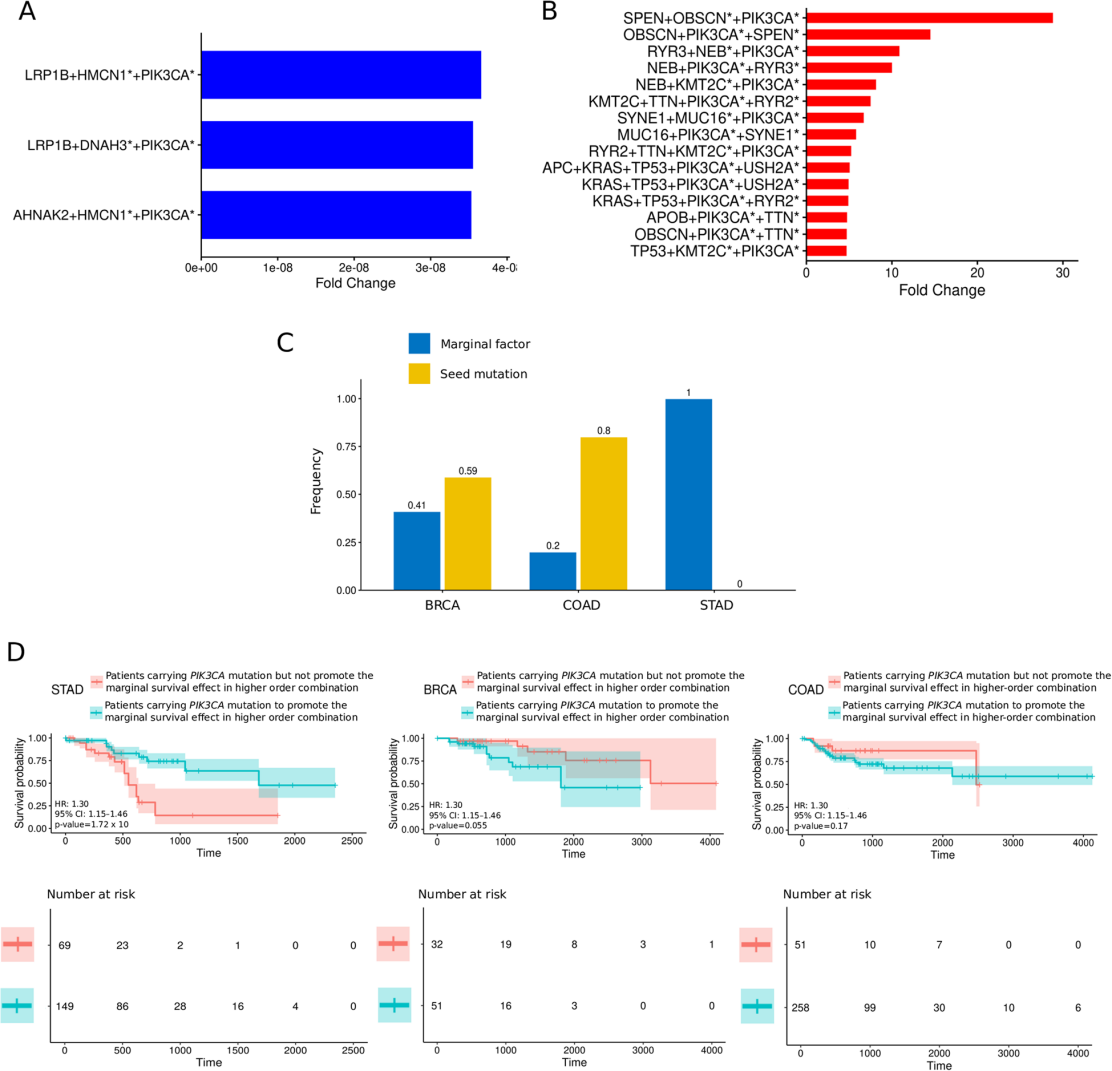
*PIK3CA* mutations promote significant marginal beneficial/adverse effects on the hazard ratio and survival probability by interacting with higher-order mutational combination elements*.* **a** The top ranked mutational combinations inducing beneficial marginal effects with a digenic marginal factor including a *PIK3CA* mutation. The x-axis denotes the fold-change in the hazard ratio induced by the marginal factor. “*” denotes the marginal factor. The y-axis denotes the species of the genetic combination. **b** The top ranked mutational combinations inducing adverse marginal effects with a digenic marginal factor including *PIK3CA* mutations. The x-axis denotes the fold-change in the hazard ratio induced by the marginal factor. “*” denotes the marginal factor. The y-axis denotes the species of genetic combination. **c** The proportion of combinations with a *PIK3CA* mutation as the marginal factor or as the seed mutation. The x-axis denotes the cancer type. The y-axis denotes the frequency value. **d** Kaplan-Meier survival curves of two patient cohorts with *PIK3CA* mutations presented in higher-order mutational combinations with or without remarkable marginal effects. The significance of the effects on survival was calculated by using the log-rank test. The x-axis denotes the survival time. The y-axis denotes the survival probability

By matching and comparing the mutational elements and HRs of homogeneous combinations with different levels of genetic order and by using the filtering standards described in Methods, the analysis identified the higher-order combinations with a *PIK3CA* mutation that could exert biological effects on the HRs across multiple cancers. We identified ~ 3238 *PIK3CA*-specific higher-order mutational combinations that produced a marginal survival effect (Fig. 2a-b, Supplementary Fig. 2A-2B, Supplementary Table 1–4). Among the higher-order combinations with a marginal effect, we found that the *PIK3CA* mutations within the BRCA and COAD samples were more likely to promote significant marginal effects as seed mutations, and the *PIK3CA* mutations within the STAD samples mostly interacted with the seed mutations to serve as the marginal factor (Fig. 2c), indicating that mutations in *PIK3CA* might be a factor initiating the remarkable HR alterations seen in the STAD samples. Strikingly, we found that the patients with STAD carrying *PIK3CA* mutations as the marginal factor or as a seed mutation had a better prognosis than patients carrying *PIK3CA* mutations that were not the marginal factor or a seed mutation (149 patients vs. 69 patients; HR 0.26, 95% CI 0.16–0.44, *p-value* = 6.177 × 10^− 8^) (Fig. 2d), suggesting that investigating those *PIK3CA* mutations that function as the marginal factor to beneficially affect survival is warranted.

### Patients carrying ***PIK3CA*** mutations can be classified into subtypes according to the frequency of their partner mutations that generate a considerable marginal effect

**Fig. 3:**
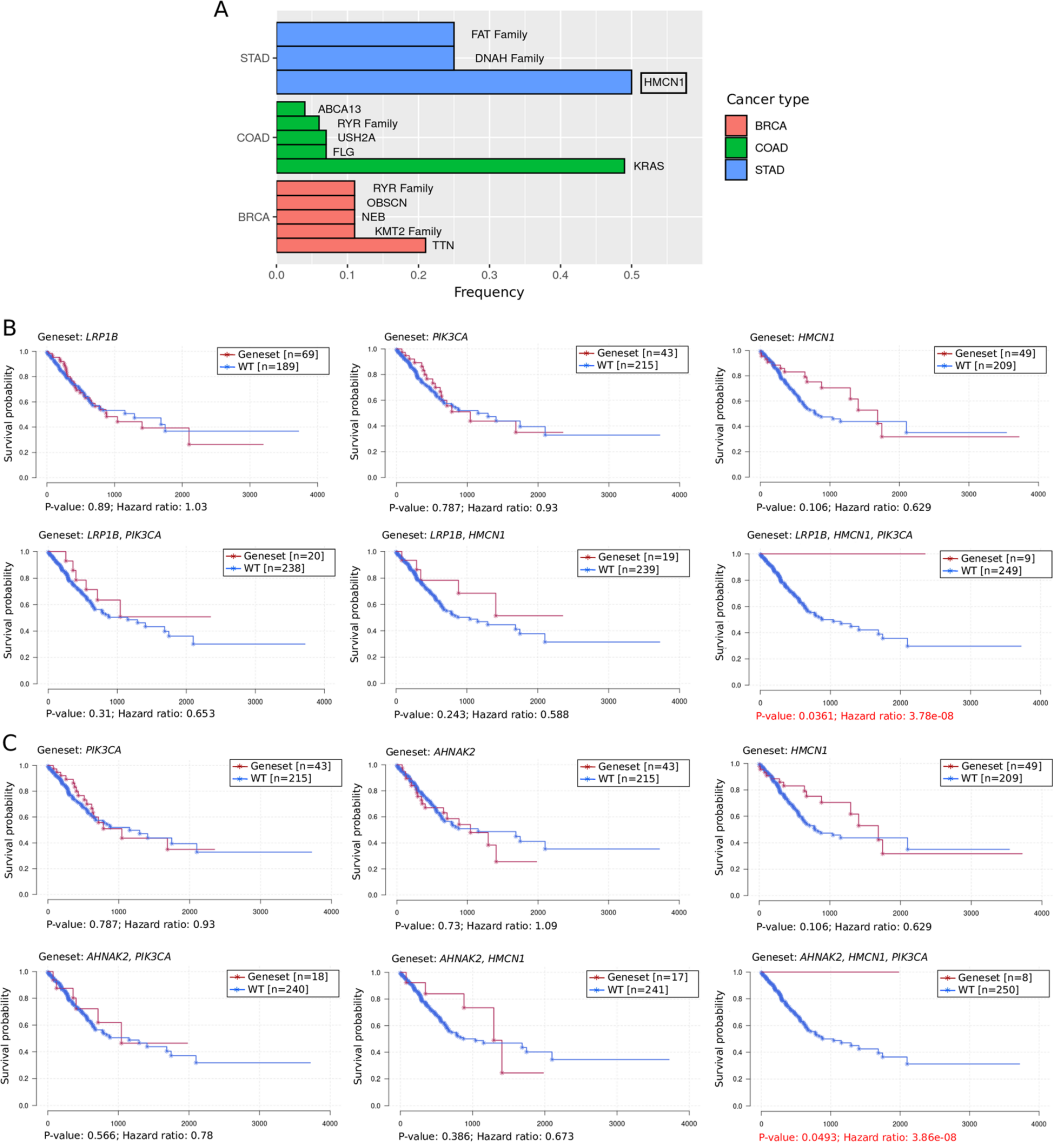
*PIK3CA + HMCN1* mutation as the marginal factor can mitigate the poor survival of the *LRP1B* and *AHNAK2* mutational subtypes by interacting within a higher-order combination. **a** The most frequently found mutational partners of *PIK3CA* mutation within all higher-order combinations in STAD, BRCA and COAD. The x-axis denotes the frequency. The y-axis denotes the cancer type. **b** Dimutation of *PIK3CA + HMCN1* produced a beneficial marginal effect on the HR compared with the *LRP1B* mutation in 60% of the STAD patient samples selected at random (the exploration set). The x-axis denotes the survival time. The y-axis denotes the survival probability. **c** Dimutation of *PIK3CA + HMCN1* produced a beneficial marginal effect on the HR compared with *AHNAK2* mutation in 60% of the STAD patient samples selected at random (the exploration set). The x-axis denotes the survival time. The y-axis denotes the survival probability

Since we discovered that *PIK3CA* mutations could remarkably influence the survival probability by interacting with other mutations in higher-order combinations and facilitating adverse or beneficial marginal effects, we were interested in which combinatorial partners might frequently interact with *PIK3CA* mutations and show a particular fixed subtype while generating the marginal effect. For this reason, we summarized the elements that interacted with *PIK3CA* mutations as digenic partners of a seed mutation or the marginal factor (Fig. 3a). Moreover, we observed that combinations consisting of mutations in *HMCN1* accounted for ~ 50% of the higher-order combinations with marginal effects containing mutations in *PIK3CA* (Fig. 3a). Intriguingly, we were particularly interested in the survival probabilities of patients carrying *LRP1B* mutations (approximately 26.7% of STAD patients carried *LRP1B* mutations) (HR = 3.78 × 10^− 8^, *p-value* = 0.0361) and *AHNAK2* mutations (approximately 16.7% of STAD patients carried *AHNAK2* mutations) (HR = 3.86 × 10^− 8^, *p-value* = 0.0493). Our analysis in another randomly selected dataset (the exploration dataset; containing 60% of samples from the total dataset) showed that the survival probabilities of these patients could be dramatically improved when *PIK3CA + HMCN1* mutations were also found with *LRP1B* and *AHNAK2* mutations in STAD patients (Fig. 3b-c). We next validated the beneficial effect of *PIK3CA* mutations compared with seed mutations of *LRP1B* and *AHNAK2* in another dataset containing 40% of samples from the overall STAD patient (Supplementary Fig. 3A-3B). The data suggested a potential clinical value of studying the molecular mechanisms underlying the trigenic interactions that could improve cancer patient survival.

### Mechanistic study of the marginal factors affecting the hazard ratio of the seed mutation in STAD patients

**Fig. 4:**
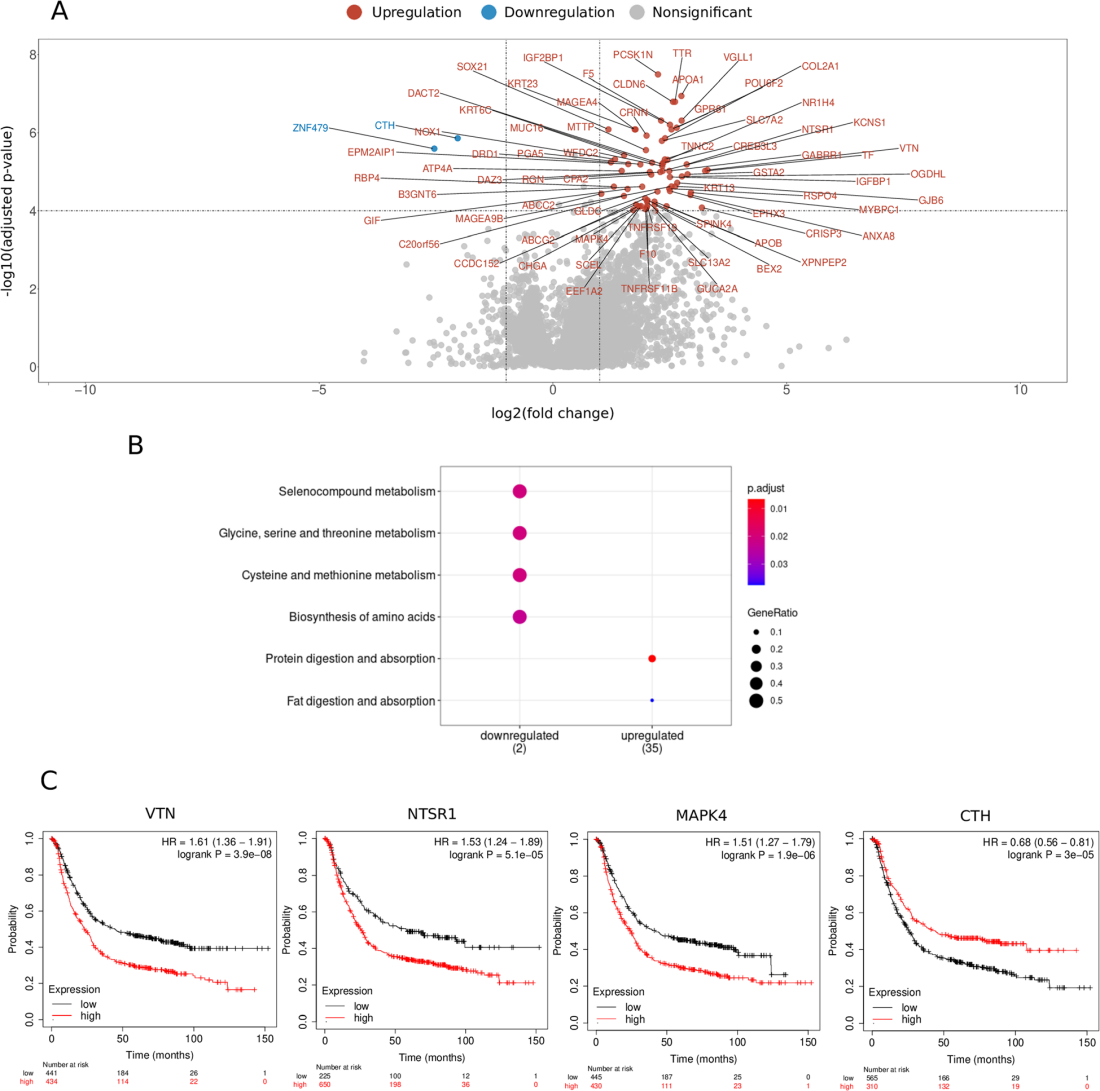
Mechanisms by which the *PIK3CA + HMCN1* mutation neutralizes the oncogenic characteristics of patients with gastric cancer carrying the *LRP1B* mutation. **a** Volcano plot of DEGs that served as the marginal effect factor in *PIK3CA + HMCN1* dimutation combinations. STAD patients with the tri-mutation of *LRP1B + PIK3CA + HMCN1* were chosen as the reference group to be compared with STAD patients with the mutation of *LRP1B* but without the di-mutation of *PIK3A + HMCN1*. Light blue dots denote downregulated genes; dark red dots denote upregulated genes; gray dots denote nonsignificant genes; **b** KEGG-based pathway enrichment. The blue color denotes a downregulation of the genes in the samples containing *LRP1B* mutation without *PIK3CA + HMCN1* compared to the samples containing *LRP1B + PIK3CA + HMCN1*; the red color denotes an upregulation of the genes in the samples with *LRP1B* mutation without *PIK3CA + HMCN1* compared to the samples with *LRP1B + PIK3CA + HMCN1.* **C** The Kaplan-Meier survival curves of gastric cancer patient cohorts presented with high expression of *VTN* > ~median value) and low expression of *VTN* (<~median value), high expression of *NTSR1* (> ~ lower quartile value) and low expression of *NTSR1* (<~lower quartile value), high expression of *MAPK4* (<~median value) and low expression of *MAPK4* (> ~ median value), high expression of *CTH* (<~median value) and low expression of *CTH* (> ~ median value), the significance of the effects on survival was calculated by using the log-rank test, *p* = 1.2 × 10^− 4^ for *VTN, p* = 5.1 × 10^− 5^ for *NTSR1*, *p* = 1.9 × 10^− 6^ for *MAPK4* and *p* = 3 × 10^− 5^ for *CTH*. The x-axis denotes the survival time. The y-axis denotes the survival probability

To further investigate the mechanistic alterations underlying the beneficial marginal effect promoted by the marginal factor (*PIK3CA + HMCN1* dimutation) compared with the seed mutation (*LRP1B* mutation), we studied the transcriptomic profiles of patients carrying *LRP1B* mutations with or without *PIK3CA* + *HMCN1* comutation. We analyzed the DEGs between samples with *LRP1B* as a seed mutation and samples with *PIK3CA* + *HMCN1* mutation as the marginal factor and found 65 upregulated genes and 2 downregulated genes that exhibited remarkable differential expression levels and statistically significant differences (Fig. 4a, Supplementary Table 5).

To better illustrate the mechanisms underlying the alterations in signaling pathways induced by combinations including *LRP1B* mutation, we next subjected these DEGs to KEGG signaling pathway analysis. We observed that the pathway terms selenocompound metabolism, cysteine and methionine metabolism, protein digestion and absorption, fat digestion and absorption were mostly enriched (Fig. 4b), implying an enrichment of pathways related to metabolic alterations, which is consistent with current knowledge about the tumor suppressor and the low-density lipoprotein (LDL) receptor functions of *LRP1B* [24]; the results suggested that the comutation of *PIK3CA* and *HMCN1* could play a role in abrogating the oncogenic metabolic signature arising from *LRP1B* mutation. The results above indicated that *PIK3CA + HMCN1* as the marginal factor might interact with *LRP1B* mutation to block oncogenic progression by regulating hazardous factors such as *vitronectin* (*VTN*), which induces cancer stemness and acts as a poor prognostic factor in gastric cancer [25] [26], *NTSR1,* which acts as a poor prognostic marker [27], and *MAPK4*, which promotes tumor progression [28], as well as facilitating beneficial survival factors such as *CTH*, which converts cystathione derived from methionine into cysteine [29] (Fig. 4c). In conclusion, these mechanism-related results could help identify appropriate corresponding drugs to prevent tumor progression as a result of the characteristics introduced by *LRP1B* mutation in STAD patients.

### Screening for potential compounds capable of exploiting the marginal beneficial effect caused by *PIK3CA* mutation in combination with comutation of *LRP1B*

Since small molecular agents to promote the beneficial effect of *PIK3CA + HMCN1* comutation over the hazardous effect of *LRP1B* mutation have yet to be developed, finding effective alternatives to produce the biological effects of these critical mutations for precise therapeutic goals is a critical task. By generating data-driven connectivity maps (CMaps) [22, 23], we aimed to select potential candidate compounds that might target emerging oncogenic pathways caused by mutations such as *LRP1B*.

To search for the most relevant compounds that could generate the RNA expression pattern induced by mutation of *LRP1B* within STAD, we input 65 significantly upregulated genes induced by the mutational combinations including *LRP1B* with or without *PIK3CA + HMCN1* as the marginal factor (Fig. 4a, Supplementary Table 5) into the CMap database, screened 47,6252 different genetic targets and treatments across different cell lines, and identified 125 items for the stomach cancer cell line AGS, in which these compounds with connectivity scores less than − 0.3 were considered a match.

**Fig. 5:**
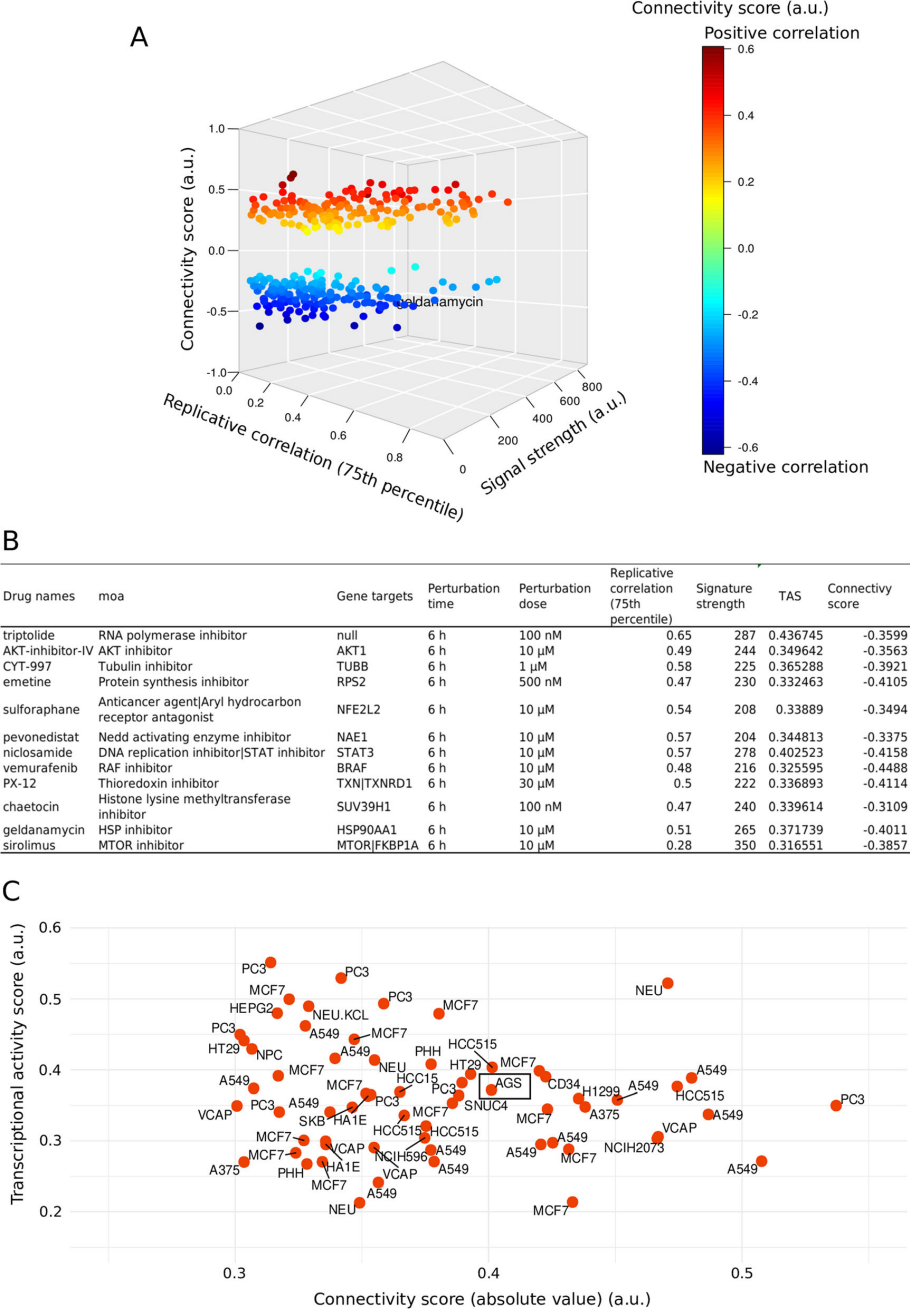
Therapeutics targeting the marginal factor *PIK3CA + HMCN1* to induce a specific RNA expression signature. **a** The strength of the signature (i.e., the magnitude of the gene expression change), the reproducibility of the signature, and the connectivity score of the candidate agents were assessed by determining the differential genetic signature caused by *LRP1B* mutation with or without *PIK3CA + HMCN1* comutation in the stomach cancer cell line AGS. The x-axis denotes the replicate correlation coefficient, which represents how consistent the replicates were in a given experiment. The y-axis denotes the signature strength, which represents the magnitude of the gene expression change elicited by the candidate compounds. The z-axis denotes the connectivity score, which demonstrates the association between the mutation combination-induced RNA signature and the small compound-induced RNA signature. A positive score represents a scenario in which the drug induced a similar signature to the mutational panel; a negative score represents a scenario in which the drug induced a signature that was opposite of the mutational panel. **b** The primary chosen drugs may specifically repress the adverse RNA signature and satisfy the CMap default standards for determining strong and reproducible drugs. **c** The transcriptional activity scores (TASs) and connectivity scores identified a wide variety of cancer cell lines, including the gastric cancer cell line AGS, that were related to and affected by treatment with the CMap-selected molecule geldanamycin

We next applied the default parameters of clue.io to obtain 12 strong and reproducible valid drugs against AGS cells, for which the signature strength should be larger than 200 and the replicative correlation (75th percentile) should be larger than 0.2. Specifically, among these 12 valid drugs, we observed that vemurafenib (connectivity score = − 0.4488, transcriptional activation score (TAS) = 0.3256), a potent MAPK inhibitor that targets aberrantly activated BRAF^V600E^ [30], geldanamycin (connectivity score = − 0.4011, TAS = 0.3717), an inhibitor of heat shock protein 90 (Hsp90) [31], and CYT-997 (connectivity score = − 0.3921, TAS = 0.3653), an inhibitor of tubulin (TUBB) [32], which is an important cytoskeleton constituent responsible for cellular motility, were chosen as the primary candidates because their signature strength (> 200) and replicative correlation (75th percentile) (> 0.2) and their reverse association with the gene signature induced by the marginal factor (<− 0.3) suggested that they could potentially promote the marginal effect seen with the *LRP1B* seed mutation (Fig. 5a-b).

Mutation of *LRP1B* without the mutational marginal factor *PIK3CA + HMCN1* influenced a wide variety of metabolic processes, including fat digestion and absorption, and famous metastasis-promoting oncogenes, such as *MAPK4* and *VTN* (Fig. 4b-c) therefore, we particularly noticed that Hsp90 could interact with AMPK and mediate acetyl-CoA carboxylase phosphorylation, in which acetyl-CoA carboxylase phosphorylation is a vital enzymatic process of fatty acid metabolism [33]. In addition, Hsp90 also serves to stabilize and correctly fold multiple significant client proteins associated with cell proliferation and cell survival [33]. We found literature-based evidence that the Hsp90 inhibitor geldanamycin could sensitize multiple cancer cell lines that are at increased levels of oxidative stress [34] and was also able to induce apoptosis in human ovarian cancer cells [35]. These results indicated that geldanamycin might be an effective tool for linking the marginal effect of higher-order interactions to the precise selection of therapeutic compounds identified by the CMap method.

Since the TAS reflects a perturbation in transcriptional activity in specific cellular contexts, we studied which cellular contexts would be relevant to geldanamycin treatment. We thus set the connectivity score threshold to − 0.3. According to the connectivity score (< − 0.3), different treatment doses of geldanamycin and different time points of geldanamycin administration were suitable for targeting the oncogenic pattern seen in gastric, colorectal, and lung cancer cell lines in which *PIK3CA + HMCN1* comutation was the marginal factor and the seed mutation was in *LRP1B*. However, the TAS should be greater than 0.2 (Fig. 5c, Supplementary Table 6). Therefore, geldanamycin might be an appropriate agent to recreate the beneficial effect induced by *PIK3CA + HMCN1* mutations in STAD patients who can precisely target the specific vulnerability of tumors carrying *LRP1B* mutations; in addition, geldanamycin may be suitable for treating other cancers with *LRP1B* mutations.

## Discussion

Through the systematic study of *PIK3CA* mutations interacting within higher-order elements, we strived to discover the crucial factors that serve as the marginal factor responsible for a remarkable survival effect and cooperate with specific mutations that constitute a higher-order combination. Our results provide unique personalized diagnostic and therapeutic insights that enable researchers to leverage the beneficial/adverse effects of *PIK3CA* mutations within the context of specific genetic combinations that influence survival probability, and they reveal the oncogenic RNA expression pattern arising from the marginal effect, which was further exploited by using CMap analysis to find potentially efficacious therapeutic agents.

Notably, *PIK3CA* mutations have been found to have a synergistic effect with mutational inactivation of PTEN in inducing drug resistance [36, 37] or in inducing poor prognosis with *EGFR/KRAS* comutations in nonsmall cell lung and colorectal cancer [38, 39]. However, it has been found that the efficacy of PI3K-targeting agents might be inadequate to treat some patients with refractory resistance even when combined with the MEK inhibitor trametinib, and this lack of efficacy might be caused by differential compensation mechanisms that confer resistance to PI3K inhibitors in *PIK3CA*-amplified head and neck cancer cells [40]. The lack of efficacy might also result from the accumulation of somatic genetic alterations as the tumor advances. Genetic or proteomic interactions with hub nodes such as *PIK3CA* might vastly increase, potentially contributing to cell resistance to these targeted therapies [41]. Given this potential, comprehensively assessing and modulating the significant hazardous effects of the current mutational contexts in patients could be the key to resolving the efficacy issue. Since *PIK3CA* has mostly been studied individually as a resistance driver gene and its potential genetic interactions with other genes or mutations at a higher-order level have mostly been ignored, we performed a large-scale systematic investigation to uncover the marginal effect of complicated higher-order interactions of *PIK3CA* across multiple *PIK3CA*-affected cancers.

Intriguingly, we found that *PIK3CA* mutations in STAD mostly acted as the marginal factor that primarily facilitated the survival effect within specific combinations of gene mutations, and most of these effects were beneficial, whereas *PIK3CA* mutations in BRCA and COAD appeared in both the marginal factor and seed mutation role and mostly induced adverse effects on survival. For example, the marginal factor dimutation of *MYCBP2 + TTN* contributed to very poor prognostic outcomes in BRCA patients carrying *PIK3CA* mutations (Supplementary Fig. 4A-4B). Determining why mutations in *PIK3CA* could interact to produce distinct prognostic outcomes in STAD patients compared to BRCA and COAD patients is another area of research worthy of future research.

Moreover, our findings also showed that the RNA expression signature underlying the marginal effects can be exploited as an alternative target; we determined the *PIK3CA*-induced RNA expression pattern inducing the marginal effects on survival and analyzed this expression signature through CMap. The analysis suggested a novel strategy to select appropriate therapeutic targets and thus increase personalized therapy precision.

Interestingly, it is worth noting that the evolutionary order of occurrence of the higher-order interacting comutations could be useful for delineating the evolution of cancer when the pioneer mutation is known. To characterize the mutation occurrence order of the *LRP1B + HMCN1 + PIK3CA* mutational combination, we followed the basic principle that the occurrence of mutations is relevant to the pathological stage [42, 43]. We then tried to analyze how the relative mutation patterns evolved with advancing tumor pathological stage. Interestingly, *LRP1B,* as the seed mutation, consistently exhibited the highest relative frequency (sunset red line) (Supplementary Fig. 5). The relative frequency of *LRP1B* + *HMCN1* dimutation drastically decreased from pathological stage I to pathological stage II (yellow line), whereas the frequencies of *PIK3CA* single mutation (light blue line) and the trimutation of *LRP1B* + *HMCN1* + *PIK3CA* greatly increased from pathological stage I to pathological stage II (dark blue line) (Supplementary Fig. 5). These results indicate that the *LRP1B* and *HMCN1* mutation and comutation of *LRP1B* + *HMCN1* might occur first at an early pathological stage, stage I, of STAD. With progression of tumor stage, the relative frequency of *PIK3CA* mutation increased, and tumors carrying dimutation of *LRP1B* + *HMCN1* might much more likely to acquire *PIK3CA* mutations in pathological stage II, which could explain why the relative frequency of dimutation of *LRP1B* + *HMCN1* severely decreased (yellow line) as the relative frequency of trimutation of *LRP1B* + *HMCN1* + *PIK3CA* increased (dark blue line). Moreover, the relative frequencies of *HMCN1* + *PIK3CA* as the marginal factor (blue line) and trimutation of *LRP1B* + *HMCN1* + *PIK3CA* as the marginal factor (dark blue line) decreased from pathological stage II to stage III and stage IV, but the relative frequencies of *LRP1B* + *HMCN1* dimutation (yellow line) and *LPR1B* + *PIK3CA* dimutation (green line) showed an increasing trend, indicating that mutations of *HMCN1* and *PIK3CA* might become mutually exclusive and might not be good for the prognosis of STAD patients at these stages.

Finally, we must clarify that the strategy to include the genes with the highest mutation rates was based on the assumption that a higher mutation rate may be shared by more patients, especially when these genes were combined. Including genes with a relatively lower rate of mutation may not be shared by sufficient patients for subsequent sufficiently valid testing. On the other hand, since enumerating and calculating the mutational combinations constituted by these single candidate genes largely expanded the magnitude of the computational load, we strived to include as many candidate genes as possible if the computational load was allowed. Thus, considering both limited factors above, we finally decided to include the top ranked 35 mutated genes in this study, but the diversity of mutations are widely existed, we believe more biological meaningful mutations would be discovered and researched in the future. Moreover, although the systematic analysis and identification of factors, including *PIK3CA,* producing a significant marginal survival effect when interacting within higher-order combinations in this research produced essential results, more works still need to be done, especially to mechanistically extend our findings beyond RNA profiling data. Future analyses require more specific research materials, such as appropriate cell and animal models, and more detailed functional analyses of potential *PIK3CA-*specific marginal effects on cellular behaviors, such as cellular communication, transformation, immunogenicity, and alteration of subcellular morphology.

## Conclusions

This research addressed the importance of studying *PIK3CA* mutation-specific effects on survival in the context of multiple mutations at the higher-order level and the value of analyzing the *PIK3CA* mutation-induced marginal effect. Moreover, this study enabled the identification of vital factors relevant to *PIK3CA* that can produce remarkable differences in survival in BRCA, COAD, and STAD. Specifically, we focused on a trigenic mutational combination, *PIK3CA + HMCN1 + LRP1B,* that promoted a beneficial survival effect in STAD patients. We next elucidated its mechanisms at the RNA expression level, and we determined the pathways enriched by the DEGs: pathways such as metabolisms of certain amino acids, protein digestion and absorption, fat digestion and absorption. Finally, we compared the specific RNA expression signature arising from the marginal effect with the RNA expression signature induced by specific small molecular compounds via CMap analysis. In addition, we assessed the corresponding compounds to determine their utility for recreating the effects created by *PIK3CA + HMCN1* mutation as the marginal factor for effective targeting of *LRP1B* mutational subtype tumors. However, the scale of the analysis of higher-order combinations could be increased by including more mutations, despite the massive computation capability needed. In the future, the mechanistic underpinnings of the marginal effect should also be studied with integrated omics data from miRNA, proteome, and methylation studies.

In summary, the systematic analysis of *PIK3CA* mutation-specific marginal effects within higher-order combinations that impact cancer patient survival allowed us to decipher complex higher-order interactions and convert these higher-order genetic interactions underlying the survival effects into practical, useful targets and drugs. These results will benefit the diagnosis and treatment of specific cancer subtypes in the future.

## Supplementary Information

**Additional file 1.**

**Additional file 2.**

**Additional file 3.**

**Additional file 4.**

**Additional file 5.**

**Additional file 6.**

## Data Availability

The Supplementary tables are available in the Synapse repository, https://www.synapse.org/#!Synapse:syn23530651. Another datasets generated during and/or analyzed during the current study are available from the corresponding author on reasonable request.

## Abbreviations

OS: Overall survival
HR: Hazard ratio
CI: Confidence interval
DEGs: Differentially expressed genes
TCGA: The Cancer Genome Atlas
BRCA: Breast adenocarcinoma
COAD: Colon adenocarcinoma
STAD: Stomach adenocarcinoma
KEGG: Kyoto Encyclopedia of Genes and Genomes
CMap: Connectivity map
TAS: Transcriptional activity score
