# Supplementary figures and images for: Identification of PIK3CA multigene mutation patterns associated with superior prognosis in stomach cancer
Source: BMC Cancer. 2021 Apr 7;21:368. doi: 10.1186/s12885-021-08115-w (PMC8028071; doi:10.1186/s12885-021-08115-w)

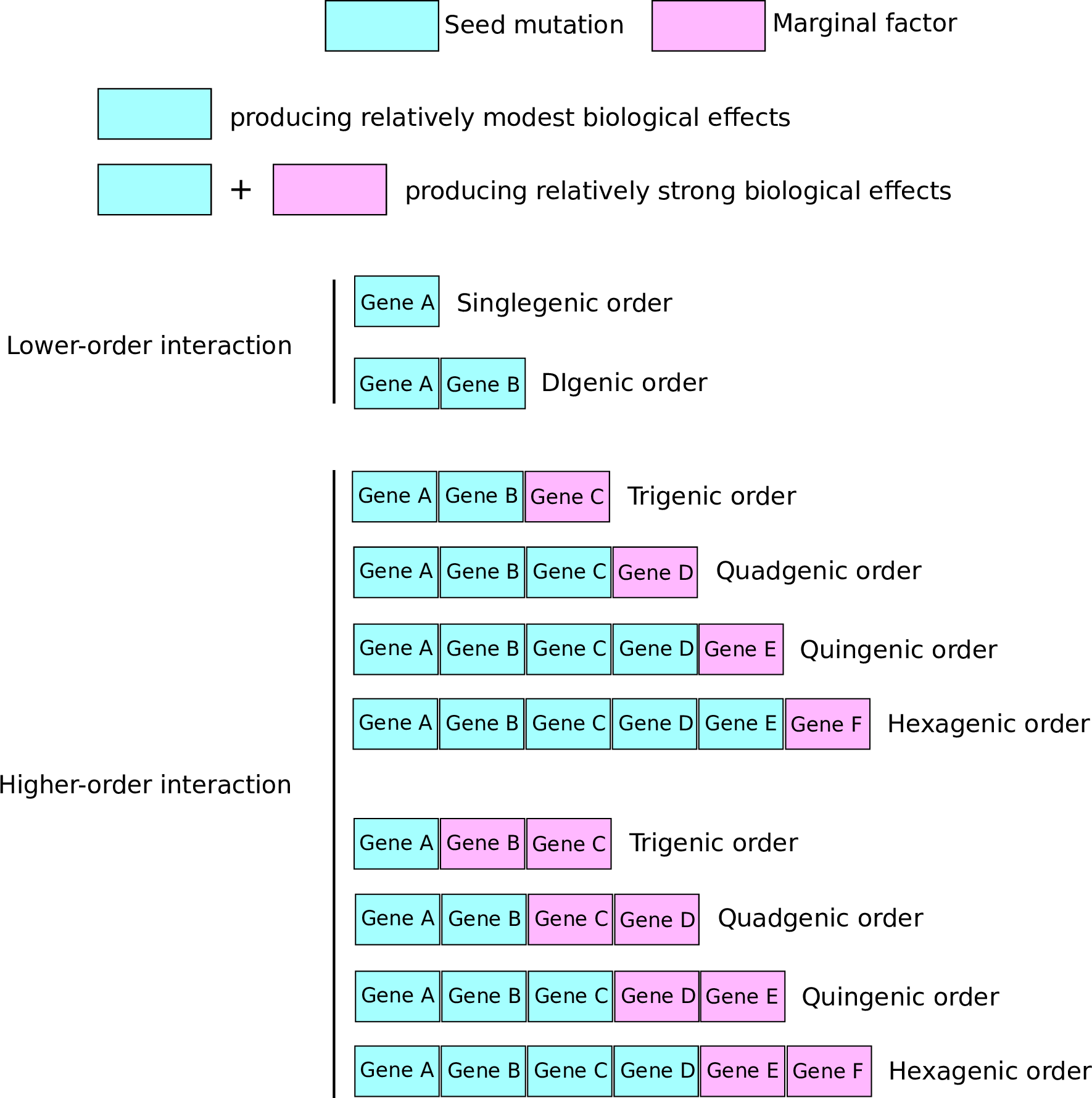

Supplement: Supplementary file 1 — Additional file 1. [file 12885_2021_8115_MOESM1_ESM.tif]

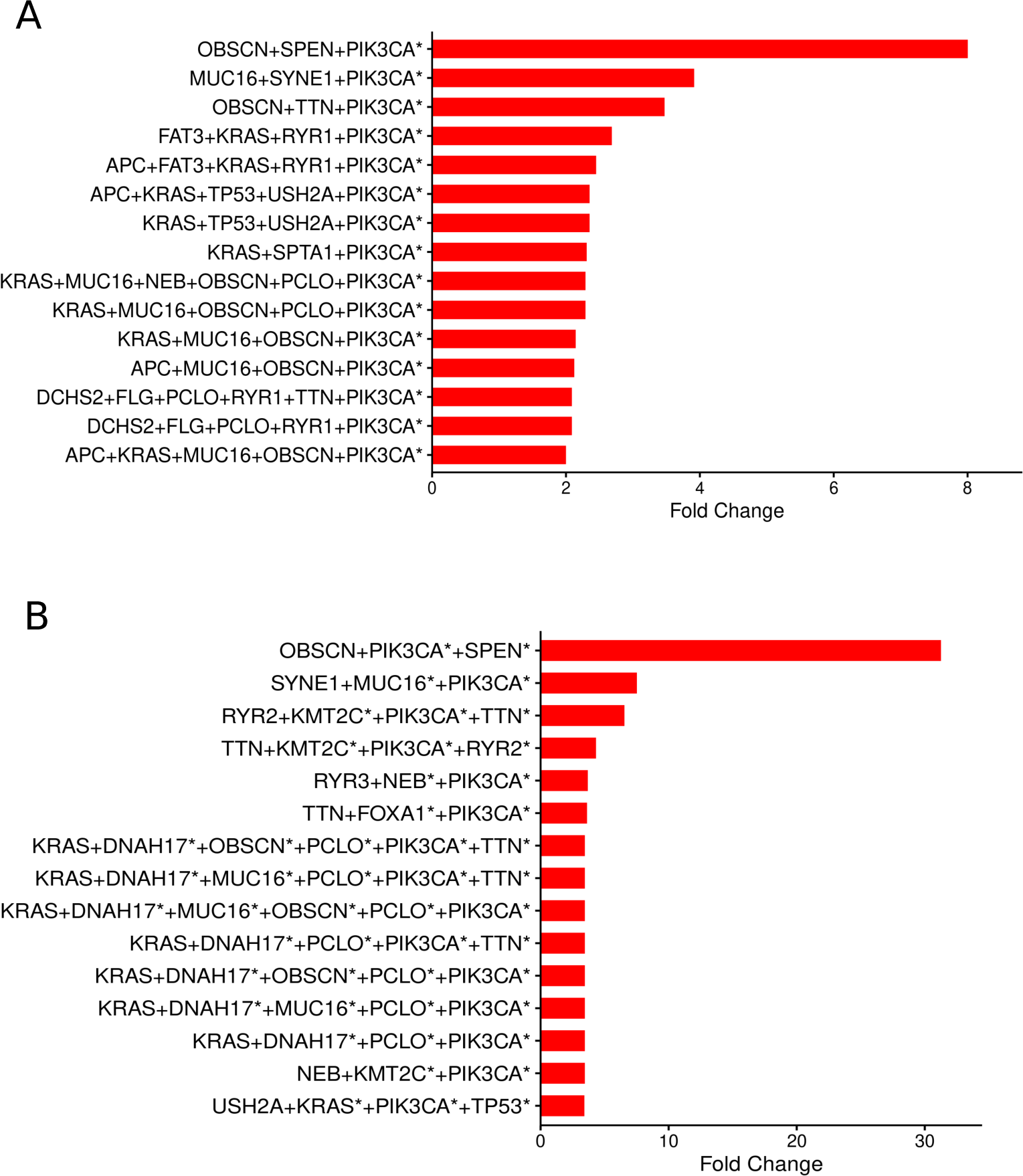

Supplement: Supplementary file 2 — Additional file 2. [file 12885_2021_8115_MOESM2_ESM.tif]

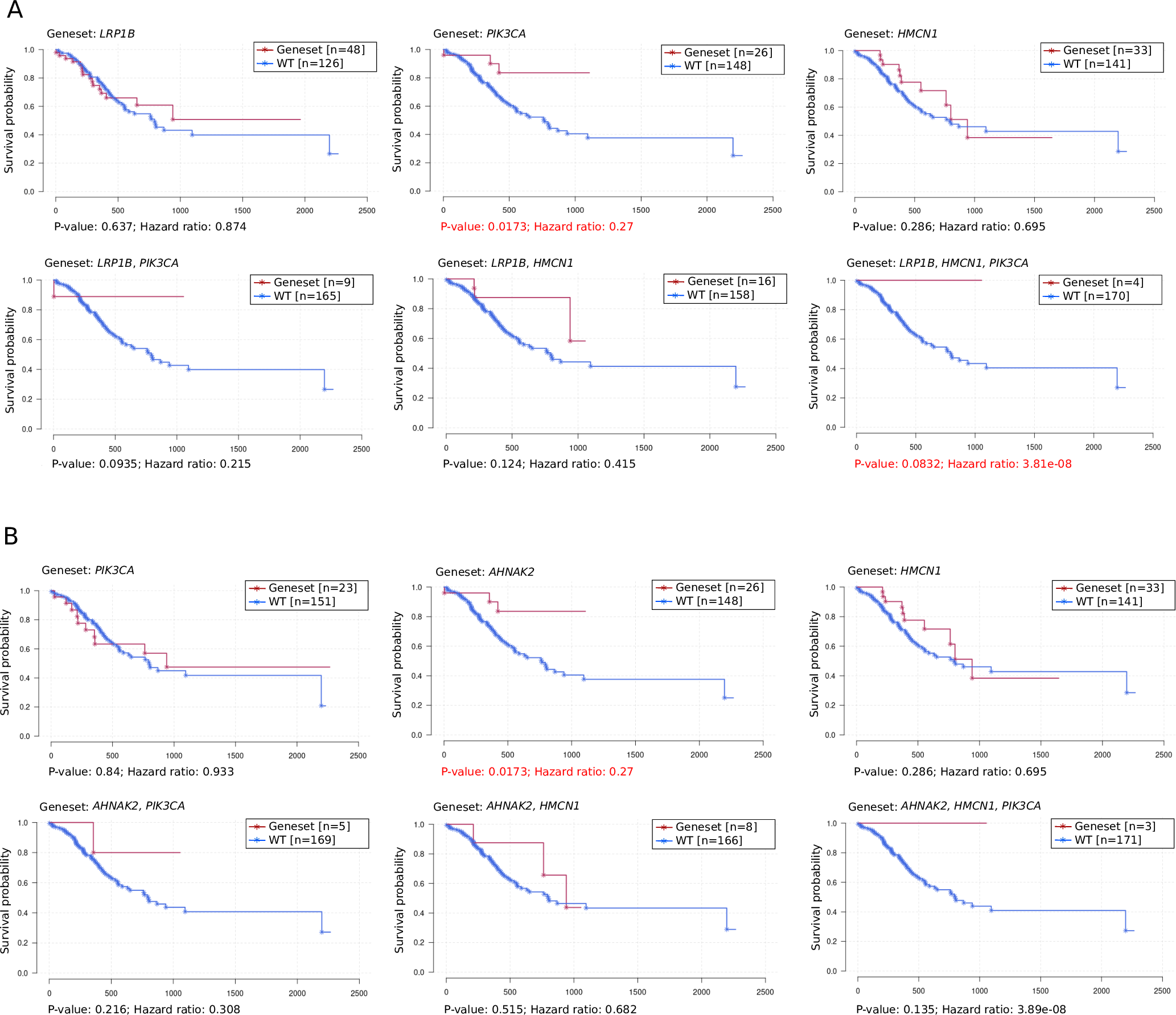

Supplement: Supplementary file 3 — Additional file 3. [file 12885_2021_8115_MOESM3_ESM.tif]

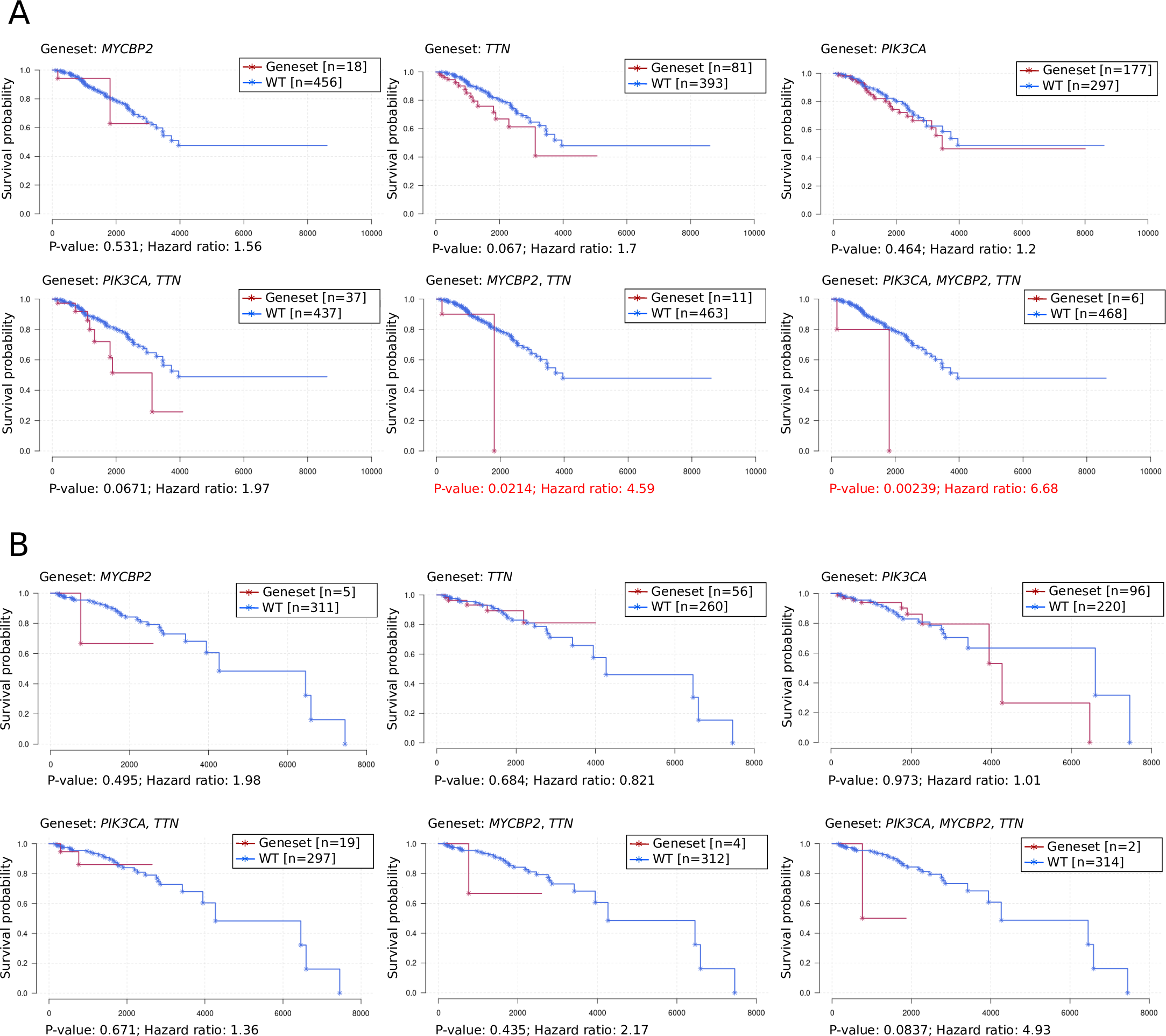

Supplement: Supplementary file 4 — Additional file 4. [file 12885_2021_8115_MOESM4_ESM.tif]

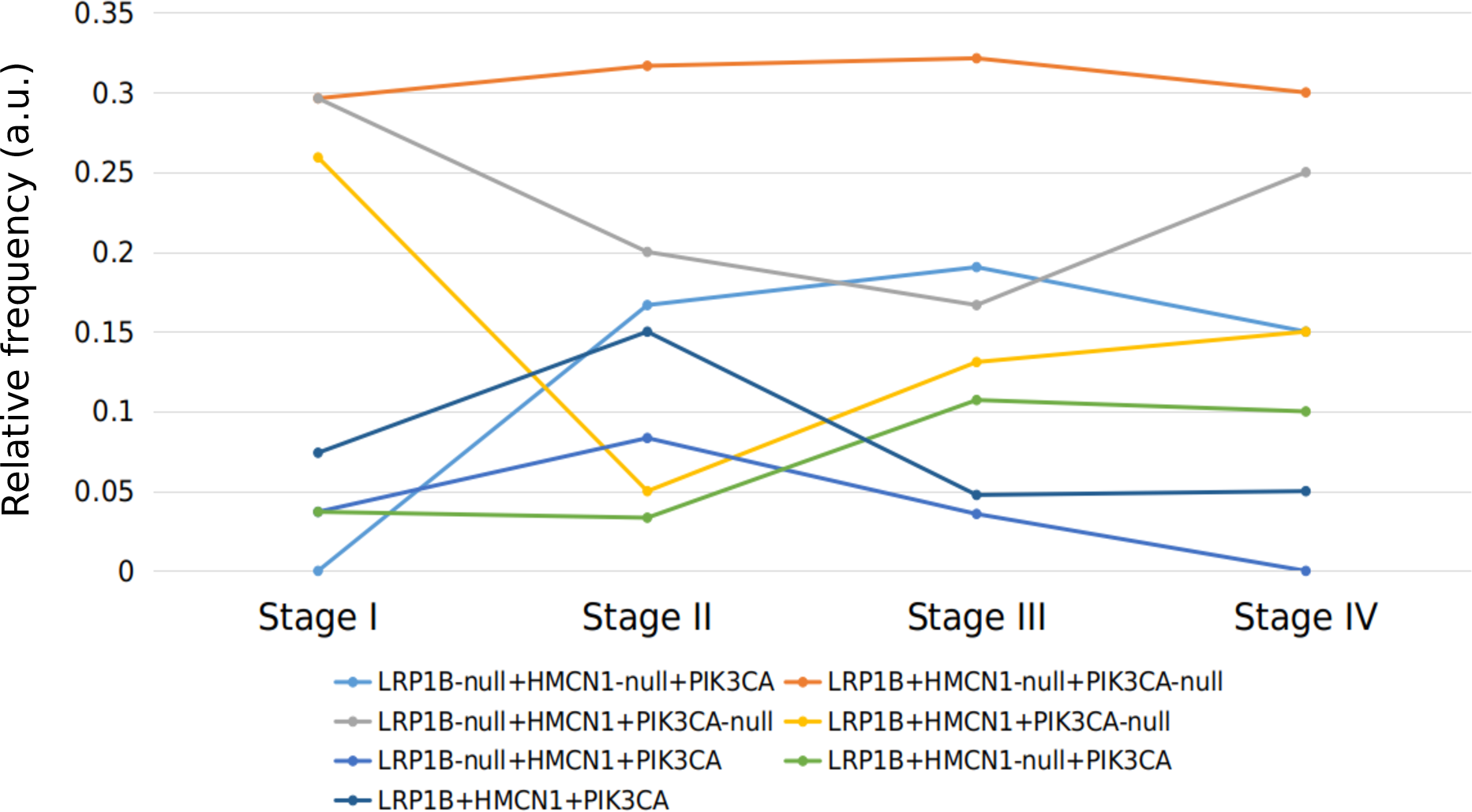

Supplement: Supplementary file 5 — Additional file 5. [file 12885_2021_8115_MOESM5_ESM.tif]
