# Supplementary material for: Identification of PIK3CA multigene mutation patterns associated with superior prognosis in stomach cancer
Source: BMC Cancer. 2021 Apr 7;21:368. doi: 10.1186/s12885-021-08115-w (PMC8028071; doi:10.1186/s12885-021-08115-w)
Supplement: Supplementary file 6 — Additional file 6. [file 12885_2021_8115_MOESM6_ESM.docx]

**Supplementary Tables**

https://www.synapse.org/#!Synapse:syn23530651
